# Anxiety symptoms and occupational stress among young Korean female manufacturing workers

**DOI:** 10.1186/s40557-015-0075-y

**Published:** 2015-11-14

**Authors:** Kang Ho Lee, Chang Ho Chae, Young Ouk Kim, Jun Seok Son, Ja-Hyun Kim, Chan Woo Kim, Hyoung Ouk Park, Jun Ho Lee, Young Saeng Jung

**Affiliations:** Department of occupational and environmental medicine, Changwon Samsung Hospital, Sungkyunkwan College of Medicine, Changwon City, Republic of Korea

## Abstract

**Background:**

The prevalence of anxiety disorders has been increasing in South Korea, with recent studies reporting anxiety disorders as the most common mental disorder among all South Korean females. Anxiety disorders, which are independent risk factors of suicidal ideation and suicide attempts, are significantly correlated with productivity loss, high medical costs, impaired work performance, and frequent worker absence, and thus are potentially serious problems affecting the health of South Korean female workers. In previous studies, anxiety disorders were shown to have a significant correlation with occupational stress. This study seeks to examine the prevalence of anxiety symptoms as well as the relationship between occupational stress and anxiety symptoms among South Korean female manufacturing workers.

**Methods:**

A structured self-reported questionnaire was administered to 1,141 female workers at an electrical appliance manufacturing plant. The questionnaire collected data on general characteristics, health behaviors, sleep quality, job characteristics (shift work, shift work schedule, and job tenure), occupational stress, and anxiety symptoms. Sleep quality was measured using the Pittsburgh Sleep Quality Index, occupational stress with the Korean Occupational Stress Scale-Short Form (KOSS-SF), and anxiety symptoms with the Korean version of the Beck Anxiety Inventory. A chi square test was conducted to determine the distribution differences in anxiety symptoms based on general characteristics, health behaviors, job characteristics, and sleep quality. A linear-by-linear association test was used to determine the distribution differences between anxietysymptoms and the levels of occupational stress. Last, logistic regression analysis was used in order to determine the association between occupational stress and anxiety symptoms.

**Results:**

The prevalence of anxiety symptoms was 15.2 %. In the multivariate logistic regression analysis that adjusted for sleep quality and general characteristics, a significantassociation was found for those with anxiety disorders; the odds ratios (OR) were significantly higher the greater the total KOSS-SF score (moderate-risk group OR=2.85, 95 % CI=1.79–4.56; high-risk group OR=5.34, 95 % CI=3.59–7.96). In addition, excluding insufficient job control, all other KOSS-SF subscales were significantly associated with anxiety symptoms, and a relatively high OR was seen in the high-risk group for job demand (OR=3.19, 95 % CI=2.27–4.49), job insecurity (OR=4.52, 95 % CI=2.86–7.13), and occupational culture (OR=4.52, 95 % CI=2.90–7.04).

**Conclusion:**

There was a significant association between anxiety symptoms and occupational stress stemming from the psychosocial work environment among these South Korean female manufacturing workers. Future longitudinal studies are needed to examine the association between the occupational stress caused by the psychosocial work environment and the incidence of anxiety disorders and anxiety symptoms. Furthermore, intervention programs that aim to address the prevalence of anxiety symptoms and improve the psychosocial work environment, especially for younger female manufacturing workers, are needed.

## Introduction

Anxiety is an emotion characterized by feelings of tension and/or worry as well as physical changes like increased blood pressure [1]. Although anxiety is a normal reaction to stress, in cases of excessive or continuous occurrence, anxiety disorders may develop [2, 3]. Anxiety disorders are a common mental disease within Western countries [4, 5]. According to the World Health Organization, the prevalence of anxiety disorders is the highest in the US, with a one-year prevalence of 18.2 %, while that in France and the Netherlands is 12.0 and 8.8 %, respectively. Among the Asian countries, this rate was relatively low, at 5.3 and 3.2 % in Japan and China, respectively [6]. In South Korea, the Epidemiological Survey of Mental Disorders was conducted in 2011 and found a one-year prevalence of 6.8 % for anxiety disorders in adults [7]. Although this prevalence is not high when compared with the rates of Western countries, a gradually increasing trend has been noted in the Survey [7]. Furthermore, anxiety disorders were found to be the most common mental disorder among Korean women, with a 1-year prevalence of 9.8 % and a lifetime prevalence of 12 % [7].

Along with depressive disorders, anxiety disorders have also been reported as a risk factor of suicidal ideation and suicide attempts [8–10]. Considering the fact that South Korea has the highest suicide rate among all of the OECD member countries (31.7 per 100,000 individuals for the total population, 43.3 per 100,000 males, and 20.1 per 100,000 females) [11, 12], the concurrent increase in anxiety disorders might explain the extremely high incidence of suicide. In addition, anxiety disorders have been reported to be significantly associated with an increase in worker absence, impaired work performance, increased medical costs, and low productivity [13–15]. Therefore, anxiety disorders might be an issue in the management of workers’ health as well as a socioeconomic issue.

According to the National Institute for Occupational Safety and Health, occupational stress is defined as the stress that occurs when the needs of the job poorly align with the abilities of the employee, available resources, and expectations of the employer, and this stress is thought to cause harmful physical and emotional responses [16]. Previous research has found that occupational stress stemming from factors in the psychosocial work environment such as work demands, insufficient job control, a lack of any reward, and low social support were significantly associated with worker anxiety symptoms or disorders [17–19].

In South Korea, research on the relationship between worker anxiety and occupational stress has found occupational stress stemming from the psychosocial work environment to be significantly related with the prevalence of anxiety symptoms in male office workers [20]. Nevertheless, few studies have investigated this relationship among manufacturing workers and/or female workers. And because women have been found to experience anxiety disorders 1.6–1.8 times more often than men do [2, 16], women are thought to be vulnerable to anxiety disorders. Therefore, research on the relationship between occupational stress and anxiety of Korean female manufacturing workers might be needed.

Accordingly, we aimed to measure the prevalence of anxiety symptoms among female workers at an electrical appliance manufacturing plant as well as examine the relationship between occupational stress and anxiety symptoms related to the psychosocial work environment. Our hypothesis is that occupational stress due to psychosocial work environment is related with anxiety symptoms of Korean female manufacturing workers.

## Materials and methods

### Subjects

From April to October of 2012, a structured, self-reported questionnaire was conducted on 1305 female manufacturing workers at the electrical appliance manufacturing plant. We excluded 164 women who did not complete the questionnaire. None of the subject answered as taking medication such as antidepressant drugs or antianxiety drugs. Accordingly, data on 1141 women were included in our analysis. The Institutional Review Board of Samsung Changwon Hospital, Changwon, Republic of Korea approved this study before implementation (no. 2014-SCMC-56-00).

### Study variables and measurements

#### General characteristics

General characteristics of all participants were examined and included age, height, weight, level of education, and marital status. Age was stratified into three groups: 10–19 years, 20–29 years, and 30–39 years. Height and weight were measured to 0.1 cm and 0.1 kg, respectively, using an automated device (GL-150, G-TECH International, Seoul, South Korea) while participants wore light clothing. Body mass index was calculated using the measured height and weight, and participants were divided into one of two groups based on their body mass indexes (<25.0 kg/m^2^ or ≥ 25.0 kg/m^2^). Education level was stratified into high school or lower and college and higher, while marital status was divided as unmarried or married.

#### Health behaviors

The health behavior of participants examined included smoking habits, drinking habits, and the frequency of regular exercise. Subjects were classified as non-smokers, ex-smokers, or current smokers. Subjects who consumed at least five glasses of alcohol more than two-times per week were classified as heavy drinkers. Regular exercise was defined as exercising at least three times per week.

#### Job characteristics

Participants were divided into categories based on their type of job in the plant. First, participants were divided into shift workers and non-shift workers. Second, job tenure was divided into those who had worked there less than 1 year, 1 to 3 years, 4 to 6 years, or seven or more years.

#### Sleep quality

Sleep quality was evaluated using a Korean version of the Pittsburgh Sleep Quality Index (PSQI), which evaluates a participant’s sleep according to seven components: sleep quality, sleep onset latency, sleep duration, sleep efficiency, sleep disturbance, use of sleeping medication, and daytime dysfunction [21]. The total score ranges from 0 to 21, with a higher score indicating a poorer quality of sleep [22]. For the present study, a PSQI score of six or higher overall was classified in the poor-quality sleep group.

#### Assessment of occupational stress

To evaluate the level of occupational stress caused by the psychosocial work environment, the Korean Occupational Stress Scale-Short Form (KOSS-SF) was used [23]. The KOSS-SF consists of 24 questions within seven subscales: job demand, insufficient job control, interpersonal conflict, job insecurity, organizational system, lack of reward, and occupational climate. Responders are rated on a 4-point Likert-type scale (1–4). A higher score indicated a relatively higher level of occupational stress. Cronbach’s alpha was used to evaluate the internal reliability of the scale and was found to be 0.52–0.82. The reference quartiles of the KOSS-SF [23] were used to evaluate the level of occupational stress based on the total KOSS-SF score and the score for each of the seven subscales. Using this reference value as the standard, the KOSS-SF scores for those in the first quartile or second quartile were classified as the low-risk group, those in the third quartile were classified as the moderate-risk group, and those in the fourth quartile were classified as the high-risk group.

#### Assessment of anxiety symptoms

The Beck Anxiety Inventory (BAI), a structured self-reported questionnaire, was used to evaluate anxiety symptoms among our participants [24]. The BAI evaluates anxiety symptoms not related to depression and is comprised of 21 questions. Responders are rated on a 4-point Likert-type scale (0–3). The total score has a range of 0–63, and the Cronbach’s alpha coefficient for internal consistency was found to range from 0.90 to 0.92 [25, 26]. The Korean version of the BAI, which was adapted by Yook and Kim [27], was used. In addition, the 22-point cut-off score proposed by Yuk and Kim [27] was used in the present study to define those with a score of 22 or higher as having anxiety symptoms.

### Analysis methods

To determine the distribution differences of anxiety symptoms according to general characteristics, health behaviors, job characteristics, and sleep quality, chi-square tests were performed. In addition, linear-by-linear association tests were conducted to determine the distribution differences of anxiety symptoms according to the level of occupational stress. Moreover, the relationship between the total KOSS-SF score with anxiety symptoms and factors significantly related with anxiety symptoms was investigated using logistic regression analysis. First, univariate logistic regression analyses were conducted to determine the influence of anxiety symptoms on each of the seven KOSS-SF subscales. Second, multivariate logistic regression analysis was conducted to adjust for variables showing a significant association in the univariate analysis. Odds ratios (OR) and 95 % confidence intervals (CI) were calculated accordingly, and the level of statistical significance was set to 0.05. All statistical analyses were conducted using IBM SPSS Statistics for Windows version 21 (IBM Corp., Armonk, NY, USA).

## Results

### Influence of general characteristics and health behaviors

The mean ± standard deviation (SD) of BAI score for the total population was 11.8 ± 9.38, with a 15.2 % prevalence of anxiety symptoms. The total age range was 18–35 years, and the mean ± SD age was 23.91 ± 3.73. The majority of subjects (75.2 %) were aged 20–29 years (18.2 % were aged 10–19 years and 5.5 % were aged 30–39 years). Anxiety symptoms did not significantly vary by age group. In addition, 13.6 % had a body mass index ≥25.0 kg/m^2^, and 22.7 % were married. High school graduates made up 89.2 % of the total population, while 10.8 % reported graduating from college or higher education. No significant difference in the distribution of anxiety symptoms was found across education, marital status, or body mass index groups. Non-smokers accounted for 67.4 % of the total population, ex-smokers for 12.8 %, and current smokers for 19.8 %. Compared with non-smokers, the distribution of anxiety symptoms was significantly higher in ex-smokers and current smokers (*p* = 0.014). In addition, 18.1 % were categorized as heavy drinkers, and this group had a significantly greater distribution of anxiety symptoms than non-heavy drinkers did (*p* = 0.006). Moreover, 20.8 % reported regularly exercising (≥3 times/week), and the distribution of anxiety symptoms did not differ across the exercise groups (Table 1).

**Table 1:** general characteristics of study population and distribution of anxiety symptoms

Variable	Number (%)	BAI^a^ score n (%)		*p*-value^*^
		<22	≥22	
Age (years)				0.493
10–19	208(18.2)	174(83.7)	34(16.3)	
20–29	858(75.2)	727(84.7)	131(15.3)	
≥ 30	75(6.6)	67(89.3)	8(10.7)	
Body mass index (Kg/m^2^)				0.718
< 25.0	986(86.4)	838(85.0)	148(15.0)	
≥ 25.0	155(13.6)	130(83.9)	25(16.1)	
Marital status				0.481
Unmarried	1018(89.2)	861(84.6)	157(15.4)	
Married	123(10.8)	107(87.0)	16(13.0)	
Educational level				0.655
≤ high school	882(77.3)	746(84.6)	136(15.4)	
≥ college	259(22.7)	222(85.7)	37(14.3)	
Smoking habit				0.014
Non-smoker	769(67.4)	669(87.0)	100(13.0)	
Ex-smoker	146(12.8)	117(80.1)	29(19.9)	
Current smoker	226(19.8)	182(80.5)	44(19.5)	
Risky drinking^b^				0.006
No	935(81.9)	806(86.2)	129(13.8)	
Yes	206(18.1)	162(78.6)	44(21.2)	
Regular exercise				0.674
< 3 times per week	904(79.2)	769(79.4)	135(78.0)	
≥ 3 times per week	237(20.8)	199(20.6)	38(22.0)	
Shift work				0.145
No	75(6.5)	68(90.7)	7(9.3)	
Yes	1066(93.5)	900(84.4)	166(15.6)	
Job tenure (years)				0.306
< 1	274(24.0)	232(84.7)	42(15.3)	
1–3	286(25.1)	237(82.9)	49(17.1)	
4–6	273(23.9)	228(83.5)	45(16.5)	
≥ 7	308(27.0)	271(88.0)	37(12.0)	
PSQI^c^ score				<0.001
< 6	450(39.4)	421(93.6)	29(6.4)	
≥ 6	691(60.6)	547(79.2)	144(20.8)	

^*^Comparison by chi-squared test

^a^Beck’s Anxiety Inventory

^b^Risky drinking: more than 2times per week and more than 5 glasses each time

^c^Pittsburgh Sleep Quality Index

### Influence of job characteristics

The vast majority of subjects (93.5 %) were shift workers, and all shift workers worked in three teams that rotated between two shifts . For job tenure, those with less than 1 year, 1 to 3 years, 4 to 6 years, and seven or more years comprised 24.0, 25.1, 23.9, and 27.0 % of the total population, respectively. The distribution of anxiety symptoms did not significantly differ across the shift work status or job tenure groups (Table 1).

### Influence of sleep quality

Having a poor quality of sleep was found to be significantly associated with having anxiety symptoms (*p* < 0.001), and 60.6 % of all participants reported having a poor quality of sleep (Table 1).

### Influence of occupational stress

The mean ± SD of total KOSS-SF score for the total population was 48.8 ± 11.2, which corresponds to the second quartile of the KOSS-SF reference value, and is lower than the average score of Korean female workers. For the scores of each KOSS-SF subscale, the average score for insufficient job control and interpersonal conflict corresponded to the third quartile of the KOSS-SF reference value, while the average score for the remaining KOSS-SF subscales corresponded to the second quartile of the KOSS-SF reference value (Table 2).

**Table 2:** Mean score of KOSS-SF^a^ and corresponding quartile for referent quartiles of KOSS-SF

KOSS-SF^a^	Mean(SD)	Reference^b^	Corresponding quartile^c^
Job demand	52.0(19.3)	58.4	2^nd^ quartile
Insufficient job control	65.6(18.8)	58.4	3^rd^ quartile
Interpersonal conflict	41.6(18.5)	33.4	3^rd^ quartile
Job insecurity	30.2(22.0)	33.4	2^nd^ quartile
Organizational system	49.8(17.4)	50.1	2^nd^ quartile
Lack of reward	54.6(20.0)	55.6	2^nd^ quartile
Occupational climate	38.4(19.1)	41.7	2^nd^ quartile
Total score of KOSS-SF	48.8(11.2)	50.1	2^nd^ quartile

^a^Korean Occupational Stress Scale-Short Form

^b^Mean score of KOSS-SF for sampled 2633 Korean female workers [24]

^c^Claassification by referent quartiles of KOSS-SF for Korean female workers

Following the data from moderate risk to high risk for the total KOSS-SF score, the distribution of anxiety symptoms significantly increased from 19.1 to 31.4 % (*p* < 0.001). For each KOSS-SF subscale, the distribution of anxiety symptoms was also found to significantly increase as the occupational stress score increased (*p* < 0.001). However, there was no significant difference in the distribution of anxiety symptoms for the insufficient job control score (Table 3).

**Table 3:** Distribution of anxiety symptoms according to occupational stress

Variable	Number (%)	BAI^a^ score N (%)		*p*-value^*^
		<22	≥22	
Job demand				<0.001
Low risk	817(71.6)	733(89.7)	84(10.3)	
Moderate risk	0(0.0)	0(0.0)	0(0.0)	
High risk	324(28.4)	235(72.5)	89(27.5)	
Insufficient job control				0.699
Low risk	510(44.7)	435(85.3)	75(14.7)	
Moderate risk	0(0.0)	0(0.0)	0(0.0)	
High risk	631(55.3)	533(84.5)	98(15.5)	
Interpersonal conflict				<0.001
Low risk	596(52.2)	529(88.8)	67(11.2)	
Moderate risk	246(21.6)	212(86.2)	34(13.8)	
High risk	299(26.2)	227(75.9)	72(24.1)	
Job insecurity				<0.001
Low risk	841(73.7)	743(88.3)	98(11.7)	
Moderate risk	186(16.3)	153(82.3)	33(17.7)	
High risk	114(10.0)	72(63.2)	42(36.8)	
Organizational system				<0.001
Low risk	726(63.6)	642(88.4)	84(11.6)	
Moderate risk	166(14.5)	136(81.9)	30(18.1)	
High risk	249(21.8)	190(76.3)	59(23.7)	
Lack of reward				<0.001
Low risk	505(44.3)	460(91.1)	45(8.9)	
Moderate risk	219(19.2)	185(84.5)	34(15.5)	
High risk	417(36.5)	323(77.5)	94(22.5)	
Occupational climate				<0.001
Low risk	555(48.6)	512(92.3)	43(7.7)	
Moderate risk	373(32.7)	304(81.5)	69(18.5)	
High risk	213(18.7)	152(71.4)	61(28.6)	
Total score of KOSS-SF^b^				<0.001
Low risk	665(58.3)	617(92.8)	48(7.2)	
Moderate risk	199(17.4)	161(80.9)	38(19.1)	
High risk	277(24.3)	190(68.6)	87(31.4)	

^*^Comparison by linear by linear association

^a^Beck’s Anxiety Inventory

^b^Korean Occupational Stress Scale-Short Form

### Association between total KOSS-SF score and anxiety symptoms, and factors associated with anxiety symptoms

The results of the logistic regression analysis revealed that as the total KOSS-SF score increased in the univariate analysis, the OR also increased significantly, and a similar pattern was seen in the multivariate analysis that adjusted for all of the general characteristics and measures of sleep quality (moderate risk group OR = 2.85, 95 % CI = 1.79–4.56; high risk group OR = 5.34, 95 % CI = 3.59–7.96). In addition, sleep quality was significantly associated with anxiety symptoms in the multivariate analysis (OR = 3.10, 95 % CI = 2.01–4.78), while smoking and heavy drinking were not significantly related with anxiety symptoms in the multivariate analysis (Table 4).

**Table 4:** Univariate and multivariate logistic regression analysis of factors affecting anxiety symptoms

Variables	Unadjusted OR			Adjusted OR^a^		
	OR^b^	95 % CI^c^	*P*-value	OR^b^	95 % CI^c^	*P*-value
PSQI^d^-score						
< 6	1.00			1.00		
≥ 6	3.82	2.51–5.81	<0.001	3.10	2.01–4.78	<0.001
Smoking habit						
Non-smoker	1.00			1.00		
Ex-smoker	1.66	1.05–2.62	0.030	1.48	0.90–2.43	0.124
Current smoker	1.62	1.09–2.39	0.016	1.47	0.96–2.25	0.077
Risky drinking						
No	1.00			1.00		
Yes	1.70	1.16–2.49	0.007	1.42	0.94–2.16	0.099
Total score of KOSS-SF^e^						
Low risk	1.00					
Moderate risk	3.03	1.92–4.80	<0.001	2.85	1.79–4.56	<0.001
High risk	5.89	3.99–8.68	<0.001	5.34	3.59–7.96	<0.001

^a^Adjusted by PSQI-score, smoking habit, risky drinking, total score of KOSS-SF

^b^Odds ratio

^c^Confidence interval

^d^Pittsburgh Sleep Quality Index

^e^Korean Occupational Stress Scale-Short Form

### Association between KOSS-SF subscales and anxiety symptoms

For both the univariate and multivariate logistic regression analyses, all KOSS-SF subscales, excluding insufficient job control, were significantly associated with anxiety symptoms. A relatively high OR was seen in the high-risk groups for the subscales job demand (OR = 3.19, 95 % CI = 2.27–4.49), job insecurity (OR = 4.52, 95 % CI = 2.86–7.13), and occupational climate (OR = 4.52, 95 % CI = 2.90–7.04) (Table 5).

**Table 5:** Univariate and multivariate logistic regression analysis of KOSS-SF^d^ subscale

Variables	Unadjusted OR		Adjusted OR^a^	
	OR^b^	95 % CI^c^	OR^b^	95 % CI^c^
Job demand				
Low risk	1.00		1.00	
Moderate risk	-	-	-	-
High risk	3.35	2.37–4.61	3.19	2.27–4.49
Insufficient job control				
Low risk	1.00		1.00	
Moderate risk	-	-	-	-
High risk	1.06	0.77–1.48	1.05	0.75–1.47
Interpersonal conflict				
Low risk	1.00		1.00	
Moderate risk	1.27	0.81–1.97	1.18	0.75–1.86
High risk	2.50	1.74–3.62	2.26	1.55–3.30
Job insecurity				
Low risk	1.00		1.00	
Moderate risk	1.64	1.06–2.52	1.54	0.99–2.40
High risk	4.42	2.86–6.83	4.52	2.86–7.13
Organizational system				
Low risk	1.00		1.00	
Moderate risk	1.68	1.07–2.66	1.61	1.01–2.58
High risk	2.37	1.64–3.44	2.32	1.58–3.40
Lack of reward				
Low risk	1.00		1.00	
Moderate risk	1.88	1.88–3.03	1.65	1.01–2.69
High risk	2.98	2.03–4.36	2.75	1.86–4.08
Occupational climate				
Low risk	1.00		1.00	
Moderate risk	2.70	1.80–4.06	2.53	1.67–3.85
High risk	4.80	3.11–7.34	4.52	2.90–7.04

^a^Adjusted by PSQI-score, smoking habit, risky drinking

^b^Odd ratio

^c^Confidence interval

^d^Korean Occupational Stress Scale-Short Form

## Discussion

This study examined the prevalence of anxiety symptoms in Korean female manufacturing workers to determine the association of anxiety symptoms with occupational stress that is thought to result from the psychosocial work environment. Our results reveal that the occurrence of anxiety symptoms is significantly related to the presence of occupational stress stemming from the psychosocial work environment.

The prevalence of anxiety symptoms among the present population was 15.2 %. In a study on the association between aircraft noise exposure and anxiety symptoms in the general Korean population aged between 30 and 70 years (mean age 60.7), 19.2 % of those in the non-noise exposure group had anxiety symptoms (a score of ≥22 for the Korean version of the BAI) [28]. The prevalence of anxiety symptoms in the previous study is higher than that reported in the present study among female workers aged between 10 and 39 years. In addition, a study that evaluated the anxiety symptoms of Korean male office workers using the Depression Anxiety Stress Scale found 19.5 % of them to have a moderate to severe level of anxiety symptoms [20]. In a study from the Netherlands on 45 Dutch office workers, the prevalence of subclinical anxiety among the women was reported as 10 % using the Hospital Anxiety and Depression Scale [18]. In China, Gao et al. [19] found the prevalence of anxiety symptoms among Chinese nurses to be 43.4 % using the Self-Rating Anxiety Scale. However, the results of these studies should be interpreted with caution since each study evaluated anxiety symptoms using different tools.

The prevalence of anxiety symptoms among female manufacturing workers of this study may not be seriously high. However, because two recent studies have suggested that the presence of anxiety symptoms is significantly related to the prevalence of suicidal ideation and suicide attempts [29, 30]. With suicide being the highest cause of death among Korean women aged between 10 and 39 years old [31], anxiety symptoms potentially can be important health problem among Korean female workers, especially 10–39 years old. Henceforth, study and management of anxiety symptoms might be needed among Korean female workers of diverse occupation, especially 10–39 years old.

The level of occupational stress stemming from the psychosocial work environment among the female manufacturing workers in the present study is lower than the average level previously reported for Korean female workers. The total KOSS-SF score as well as the scores for job demand, job insecurity, organizational system, lack of reward, and occupational climate were lower than the average score previously reported for Korean female workers. However, the average scores for insufficient job control and interpersonal conflict were higher than the average score previously reported for Korean female workers. Among the total population, 55 % showed scores that fell into the high-risk category(the fourth quartile of the KOSS-SF reference values) for insufficient job control, which was also found to be the greatest cause of occupational stress. Because the work-related responsibilities of these female workers are relatively simple and the majority (93.6 %) is shift workers, it might be difficult for them to adjust their workload or work schedule, thus creating high levels of occupational stress.

We found a significant association between anxiety symptoms with the total KOSS-SF score and all KOSS-SF subscales besides insufficient job control, even after adjustment for general characteristics and sleep quality. In a study by Park et al. [20] on Korean male office workers, a significant association was observed between anxiety symptoms with the total KOSS-SF score and all KOSS-SF subscales besides insufficient job control and job insecurity, a finding that is similar to the results of the present study. However, the observation of a significant association between job insecurity and anxiety symptoms in the present study was lacking in the study of Park et al. [20]. In a longitudinal study by Plaisier et al. [32], no significant association between job security and incidence of anxiety disorders in men was found; however, in women, job security had a protective effect against the later incidence of anxiety disorders. Therefore, sex-specific differences might explain the discrepancies noted between these studies.

In this cross-sectional study, a relatively high OR was seen in the high-risk groups for the subscales job demand (OR = 3.19, 95 % CI = 2.27–4.49), job insecurity (OR = 4.52, 95 % CI = 2.86–7.13), and occupational climate (OR = 4.52, 95 % CI = 2.90–7.04). In two longitudinal studies, job demand was found to be significantly associated with GAD or anxiety symptoms among female workers [17, 33]. Also job insecurity was found to be significantly associated with anxiety disorders of female workers in a longitudinal study [32]. However few studies have investigated this relationship in longitudinal study design among Korean female workers. Factors related to stress resulting from the occupational climate such as an authoritative and hierarchical workplace, irrational communication, and the uncomfortable atmosphere of company dinners, all of which are commonplace in South Korea, is evaluated in the section of occupational climate. In the cross-sectional study Among Korean male office workers, the occupational climate was found to be significantly associated with anxiety symptoms [20]. However, few studies have attempted to investigate the possible association between the very hierarchal workplace climate in South Korea and anxiety, especially among Korean female workers. Accordingly, longitudinal study which investigate the relationship between anxiety and job demand, job insecurity, occupational climate among Korean female workers is needed.

The level of occupational stress caused by insufficient job control for subjects of the present study was higher than the average score previously reported for Korean female workers. Even though 55 % of these workers fell into the high-risk group (the fourth quartile of the KOSS-SF reference values), there was no significant association with anxiety symptoms. In the longitudinal study of Andrea et al. [33], decision latitude was not significantly associated with the future incidence of subclinical anxiety symptoms. In addition, in other two longitudinal studies, decision latitude was not significantly related with the occurrence of generalized anxiety disorder in women [17] or with the occurrence of anxiety disorders [32]. The results of our study correspond with those of previous longitudinal studies.

In a cross-sectional study of Korean male office workers and in a cross-sectional study of Italian radiologists, relational conflict among coworkers or superiors was significantly associated with anxiety symptoms [20, 34]. In our study, interpersonal conflict displayed a significant association with anxiety symptoms, confirming these previous results. However, in the longitudinal study by Andrea et al. [33], conflict with coworkers or superiors was not significantly related with the future incidence of subclinical anxiety symptoms; these results differed from those of this study and another cross-sectional study [33].

For the Korean male office workers, there was a significant association reported between the organizational system and anxiety disorders [20], and similar results were found in the female manufacturing workers of our study. For the Korean male office workers and Italian radiologists from the two previous studies mentioned above, a significant association between lack of reward and anxiety symptoms was also reported [20, 34]; similar results were confirmed in the present study.

Aside from occupational stress, poor sleep quality was significantly associated with anxiety symptoms in the multivariate logistic regression analysis. Sleep quality was poor for 60.6 % of our subjects, and the majority (93.5 %) of our subjects were shift workers. The association between shift work and sleep disturbance is well known [35]. Therefore, it is of concern that the ratio of workers with poor sleep quality is relatively high. In previous studies, insomnia was associated with clinically significant anxiety or anxiety disorders and was significantly associated with the future incidence of anxiety disorders [36–40]. Likewise, in our study, those with a poor quality of sleep were significantly more likely to have anxiety symptoms than those who did not have poor quality of sleep. Therefore, our results are similar to those of previous studies.

Our study has the following limitations. First, a survey was used to estimate the prevalence of anxiety symptoms as opposed to the use of diagnostic criteria to clinically diagnose these anxiety disorders. Second, the temporal relationship between anxiety symptoms and occupational stress caused by the psychosocial work environment cannot be established from the present study. Thus, future longitudinal studies are needed that employ diagnostic criteria to define anxiety disorders as well as determine whether a temporal relationship exists. Third, the use of a self-reported questionnaire can lead to biases based on the individual subjectivity of the responders. Fourth, we did not investigate whether other individual and/or familial factors are associated with anxiety symptoms [41]. Finally, the limited age range limits the generalizability of our results to the general Korean female workers.

Despite these limitations, this study is important because it confirms the significant association between occupational stress and anxiety symptoms as well as draws attention to this issue that affects Korean female manufacturing workers, especially 19–35 years old. Large study population is also strong point of this study. Anxiety disorders are the most common mental disorder among Korean women, with an increasing prevalence. Considering the lack of research on the association of occupational stress with anxiety symptoms or anxiety disorders among Korean female workers, future studies are needed on subjects from a diverse range of occupations and age groups. Furthermore, there might be a need for intervention programs [42–44] that appropriately manage the psychosocial work environment and factors associated with anxiety symptoms and anxiety disorders, especially among young female manufacturing workers.
